# Syrup of Hydrocyanic Acid

**Published:** 1847-09

**Authors:** 


*Syrup of Hydrocyanic Acid.*—At the request of several medical practitioners a very elegant syrup of prussic acid has been devised by Dr. Reich, affording an eligible means of administering that potent substance in a certain dose. He prepares it by adding to a syrup of sweet almonds a definite quantity of amygdaline. His recipe is as follows:—Take of sweet almonds two ounces; immerse them for the space of a night in cold distilled water, and in the morning remove the skin by the pressure of the finger and thumb; then pound the almonds in a deep mortar, adding two ounces of the purest sugar. Pound together in a mortar, either of porcelain or marble; then by degrees add distilled water two ounces, and strain, with the application of slight force. To this emulsion add sugar of the purest kind two ounces, and promote the solution of the sugar by mixture alone, heat being avoided. To four ounces of this syrup add seventeen grains of amygdaline, and rub together in a porcelain mortar. Much of the syrup need not be kept ready prepared, as its extemporaneous formation is so easy. An ounce contains a quarter of a grain of real prussic acid.—*Ibid. from Buch. Rep.*

